# Bovine serum albumin nanoparticles as controlled release carrier for local drug delivery to the inner ear

**DOI:** 10.1186/1556-276X-9-343

**Published:** 2014-07-09

**Authors:** Zhan Yu, Min Yu, Zhibao Zhang, Ge Hong, Qingqing Xiong

**Affiliations:** 1 Department of ENT, The Second Artillery General Hospital of Chinese People's Liberation Army, 16 Xinjiekou Outer Avenue, Beijing 100088, People's Republic of China; 2 Department of Cell Biology, Key Laboratory of Cell Biology, Ministry of Public Health, College of Basic Medicine, China Medical University, 92 Beier Road, Shenyang 110001, People's Republic of China; 3 Institute of Biomedical Engineering, Chinese Academy of Medical Sciences & Peking Union Medical College, The Key Laboratory of Biomedical Material of Tianjin, Tianjin 300192, People's Republic of China

**Keywords:** Bovine serum albumin, Nanoparticle, Controlled release, Drug delivery, Round window membrane, Inner ear

## Abstract

Nanoparticles have attracted increasing attention for local drug delivery to the inner ear recently. Bovine serum albumin (BSA) nanoparticles were prepared by desolvation method followed by glutaraldehyde fixation or heat denaturation. The nanoparticles were spherical in shape with an average diameter of 492 nm. The heat-denatured nanoparticles had good cytocompatibility. The nanoparticles could adhere on and penetrate through the round window membrane of guinea pigs. The nanoparticles were analyzed as drug carriers to investigate the loading capacity and release behaviors. Rhodamine B was used as a model drug in this paper. Rhodamine B-loaded nanoparticles showed a controlled release profile and could be deposited on the osseous spiral lamina. We considered that the bovine serum albumin nanoparticles may have potential applications in the field of local drug delivery in the treatment of inner ear disorders.

## Background

Inner ear disorders, including sensorineural hearing loss (SSHL), commonly occur in clinics. The traditional systemic therapies are almost ineffective due to the blood-labyrinth barrier, which prevents the transport of drugs from the serum. Local drug delivery, especially intratympanic injection, has become popular for two decades because of its efficiency and safety. The round window membrane (RWM) is a semipermeable membrane between the middle and the inner ear, through which particles less than 3 μm in diameter could penetrate.

Local drug delivery to the inner ear by intratympanic injection was first described by Schuknecht in 1956 in the treatment of Ménière's disease [1]. In 2006, Kopke et al. reported a significant hearing improvement of patients with sudden sensorineural hearing loss after methylprednisolone administration locally [2].

Although intratympanic injection is easy to perform in the clinic, the loss of drug through the Eustachian tube becomes the obstacle to treat inner ear disorders efficiently. Thus, hydrogel- and particle-based vehicles (or carriers) have been investigated recently for sustained and prolonged drug supply. In 1998, Balough et al. described that the local injection of a fibrin-based sustained release vehicle impregnated with gentamicin allowed for a prolonged effect without absorption in the untreated ear or blood [3]. Horie et al. reported that drug-loaded polylactic/glycolic acid (PLGA) microparticles were capable of delivering lidocaine into the cochlea in a sustained manner [4]. The PLGA nanoparticles were found to be distributed throughout the inner ear after application on the RWM of chinchilla [5]. Moreover, Tan et al. demonstrated that brain-derived neurotrophic factor encapsulated in nanoporous poly(l-glutamic acid) particles could be released in a sustained manner with maintained biological activity and efficiently rescue primary auditory neurons in the cochlea of guinea pigs with sensorineural hearing loss [6]. Nowadays, nanoparticles have received much more interest for the treatment of inner ear diseases for their drug loading and sustained release capacity.

Various methods such as desolvation, emulsion, template, microfluidic, mechanical stretching, and self-assembly have been reported to prepare protein-based particles or capsules [7-11]. In this study, we demonstrated that bovine serum albumin (BSA) can form nanospheres by desolvation method and can be used for local drug delivery.

BSA is a natural protein able to form complexes in various shapes. This protein is biocompatible, biodegradable, nontoxic, and nonimmunogenic. Due to these features, albumin particles are a good system for drug and antigen delivery [11-14]. To the best of our knowledge, there have been no reports of local delivery of drug-loaded albumin particles into the inner ear. Here, we illustrate a method for creating sphere-shaped BSA nanoparticles (BSA-NPs) with biocompatibility in high yield. A model drug, rhodamine B (RhB), was loaded onto the BSA-NPs for drug loading capacity, release, and *in vivo* studies. *In vivo* biodistribution suggested that the RhB released as well as the RhB-loaded BSA-NPs (RhB-BSA-NPs) tended to accumulate and penetrate through the RWM of guinea pigs. Therefore, the BSA-NPs would be prospectively considered as controlled release carriers for local drug delivery in the treatment of inner ear disorders.

## Methods

### Materials, mice, and cell culture

BSA and RhB were purchased from Sigma-Aldrich (St. Louis, MO, USA). Cell counting kit-8 (CCK-8) was purchased from Dojindo Molecular Technology Inc. (Shanghai, People's Republic of China). Ultrapure water used in all experiments was produced by Milli-Q synthesis system (Millipore Corp., Billerica, MA, USA). L929 mouse fibroblast cells (obtained from the Cancer Institute of the Chinese Academy of Medical Sciences, People's Republic of China) were cultured in Dulbecco’s modified Eagle’s medium (DMEM) (HyClone, Thermo Scientific Inc., Waltham, MA, USA) containing 10% fetal bovine serum (FBS) at 37°C with 5% CO2. Guinea pigs weighing 250 ~ 300 g were purchased from the Tianjin Experimental Animal Center, People's Republic of China, and had free access to food and water. Animal study protocols were approved and performed in accordance with the recommendations in the Guide for the Care and Use of Laboratory Animals.

### Preparation of BSA-NPs and RhB-BSA-NPs

BSA-NPs were prepared by the desolvation method. Briefly described, 100 mg of BSA was dissolved in 1 ml of sodium chloride solution (10 mM). Then, 8.0 ml of ethanol was added dropwise into the BSA solution under magnetic stirring (400 rpm) at room temperature. Subsequently, the as-prepared BSA-NPs were cross-linked with 0.2% glutaraldehyde (GA) for 24 h or denatured at 70°C for 30 min. BSA-NPs (50 mg) were incubated with certain amounts (5, 10, 15, 17.5, and 20 mg) of RhB for 2 h in the preparation of RhB-BSA-NPs. The particles were centrifuged and washed with ultrapure water.

### Characterization of the BSA-NPs

The morphological characteristics were determined by transmission electron microscopy (TEM, JEOL, JEM-100CXII, Akishima-shi, Japan), scanning electron microscopy (SEM, ZEISS SUPRA 55VP, Oberkochen, Germany), and confocal laser scanning microscopy (CLSM, FV-1000, Olympus Corporation, Shinjuku-ku, Japan). For TEM, a drop of diluted suspension of BSA-NPs was placed on the copper grid and the air-dried specimen was observed. For SEM, a drop of diluted suspension was deposited on a silicon wafer. The air-dried sample was coated with gold and observed. RhB-BSA-NPs were observed by CLSM at an excitation wavelength of 555 nm and an emission wavelength of 580 nm.

The BSA-NPs were dispersed in ultrapure water at a concentration of 0.1 mg/ml. The particle size and zeta potential determinations were performed by using a Malvern particle size analyzer (Zetasizer Nano-ZS, Malvern, UK).

### Drug loading capacity and encapsulation efficiency

BSA-NPs (50 mg) were incubated with RhB (5 ~ 20 mg) for 2 h. After washing with ultrapure water, the supernatants were collected and analyzed for residual drug concentration by UV-vis analysis.

The drug loading capacity and encapsulation efficiency were calculated as follows:

$$\begin{array}{ll} \;\text{Drug}\;\text{loading}\;\left(w/w\%\right) & =\text{Amount}\;\text{of}\;\text{RhB}\;\text{in}\;\text{BSA}‒\text{NPs}\\ & \;/\text{Amount}\;\text{of}\;\text{BSA}‒\text{NPs}\times 100 \end{array}$$

Encapsulation efficiency (*w* / *w*%) = amount of RhB in BSA-NPs/RhB initially added × 100

### *In vitro* drug release behavior

The assay was evaluated in a standard static diffusion cell at a speed of 100 rpm in a shaker at 37°C. The amount of RhB was evaluated using UV-vis spectrometer (560 nm). The amount of RhB released was evaluated at a series of time points, and the release curve was made accordingly.

### Cell biocompatibility assay

Cells were seeded in 96-well plates at a density of 1,000 cells/well. BSA-NPs with GA fixation (NP-GA) or heat denaturation (NP-H) were added to each well for a 24-h incubation. Cell viability was determined by CCK-8 assay. Untreated cells served as the control. The morphology of L929 cells in each group was also observed by using a phase contrast microscope.

### *In vivo* assay

Guinea pigs were killed to sample the acoustic bullae (including the RWM). The acoustic bullae were placed in the solution of BSA-NPs and shaking for 30 min at 37°C. The air-dried specimens were observed by SEM.

The penetration of RhB released from the RhB-BSA-NPs was evaluated by live images and microscopes. Guinea pigs were anaesthetized and the RWMs were exposed. The heat-denatured RhB-BSA-NPs and RhB dispersed in PBS were injected slowly into the bullae of the right and left ear, respectively. The left ear injected with RhB solution was the control. *In vivo* imaging system (Caliper IVIS imaging system, PerkinElmer, Waltham, MA, USA) was used to trace the particles at time points of 0 and 72 h. The RWM was then imaged by fluorescence microscopy and SEM to observe the distribution of RhB and BSA-NPs.

### Statistical analysis

The statistical data was presented as the mean value and standard deviation. The analysis of *t* test was used in SPSS 12.0 to determine significant differences between groups, and *P* values less than 0.05 were considered statistically significant.

## Results and discussion

### Morphology of BSA-NPs

**Figure 1:**
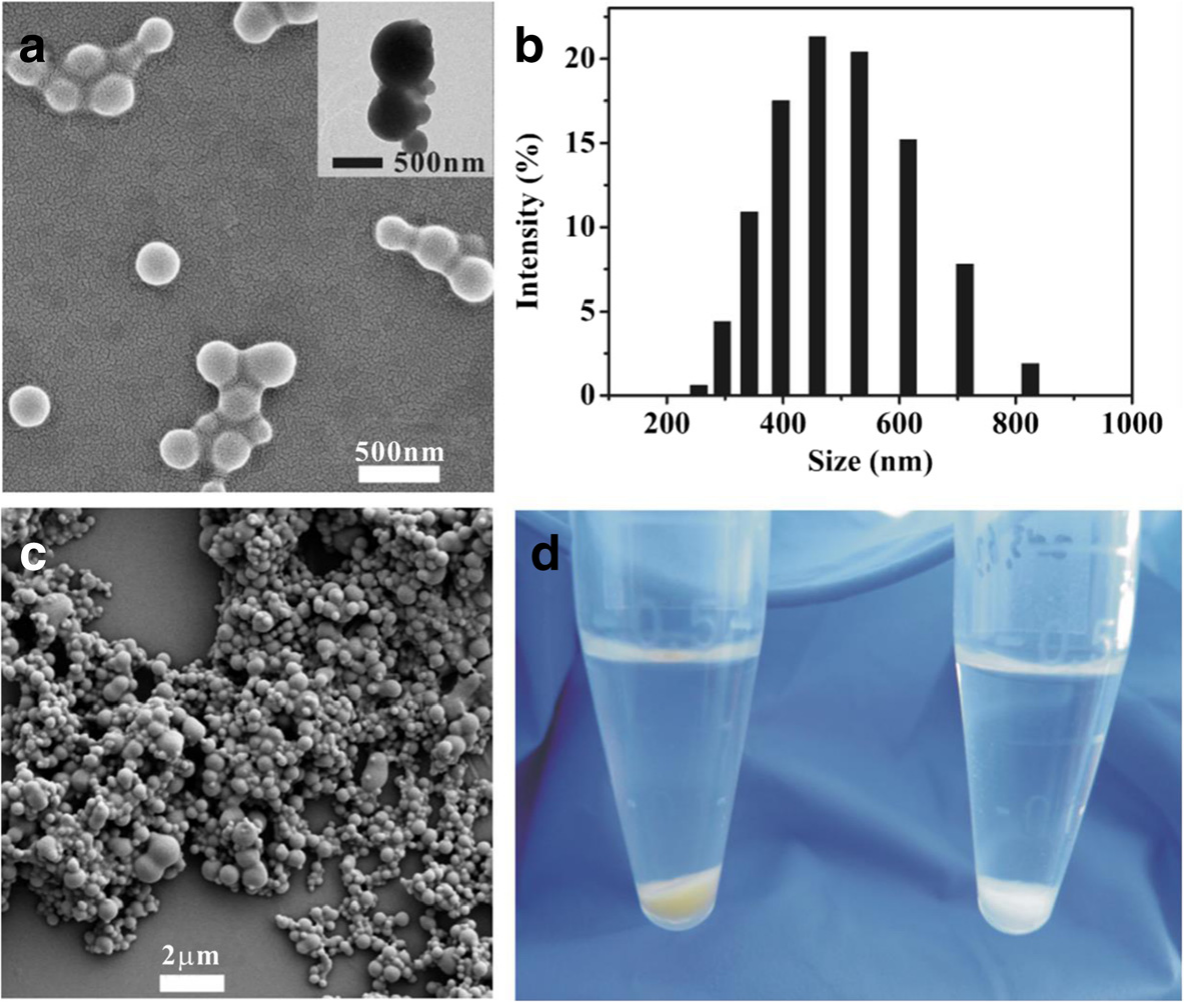
**Morphology of BSA-NPs with heat denaturation and GA fixation.** SEM/TEM images of BSA-NPs with heat denaturation **(a)** and GA fixation **(c)** are shown. The size distribution of NP-H evaluated by DLS is shown in **(b)**. The difference between the two kinds of NPs is shown in **(d)**.

BSA-NPs were prepared by the desolvation method in high yield (about 95%). Figure 1a and its inset display that BSA-NPs with heat denaturation occur as solid spheres with smooth surface. The size of these spheres determined by dynamic light scattering (DLS) varied from 255 to 825 nm (Figure 1b). The mean value was 492 nm and was larger than the size of 238 nm measured by SEM (analyzed by ImageJ 1.44 software) due to the shrinkage of the particles during dehydration. The difference between SEM and DLS is consistent with the previous literatures [8,15].As shown in Figure 1c, BSA-NPs with GA fixation were also sphere-shaped with a mean diameter of 320 nm. Therefore, we can conclude that the morphology of BSA-NPs shows no obvious difference in shape even if treated by either heat or GA. However, there was little difference between the particles viewed by the naked eye - the colors of precipitates were yellow (Figure 1d, left) and milk white (Figure 1d, right), respectively.

### Drug loading and release study

**Figure 2:**
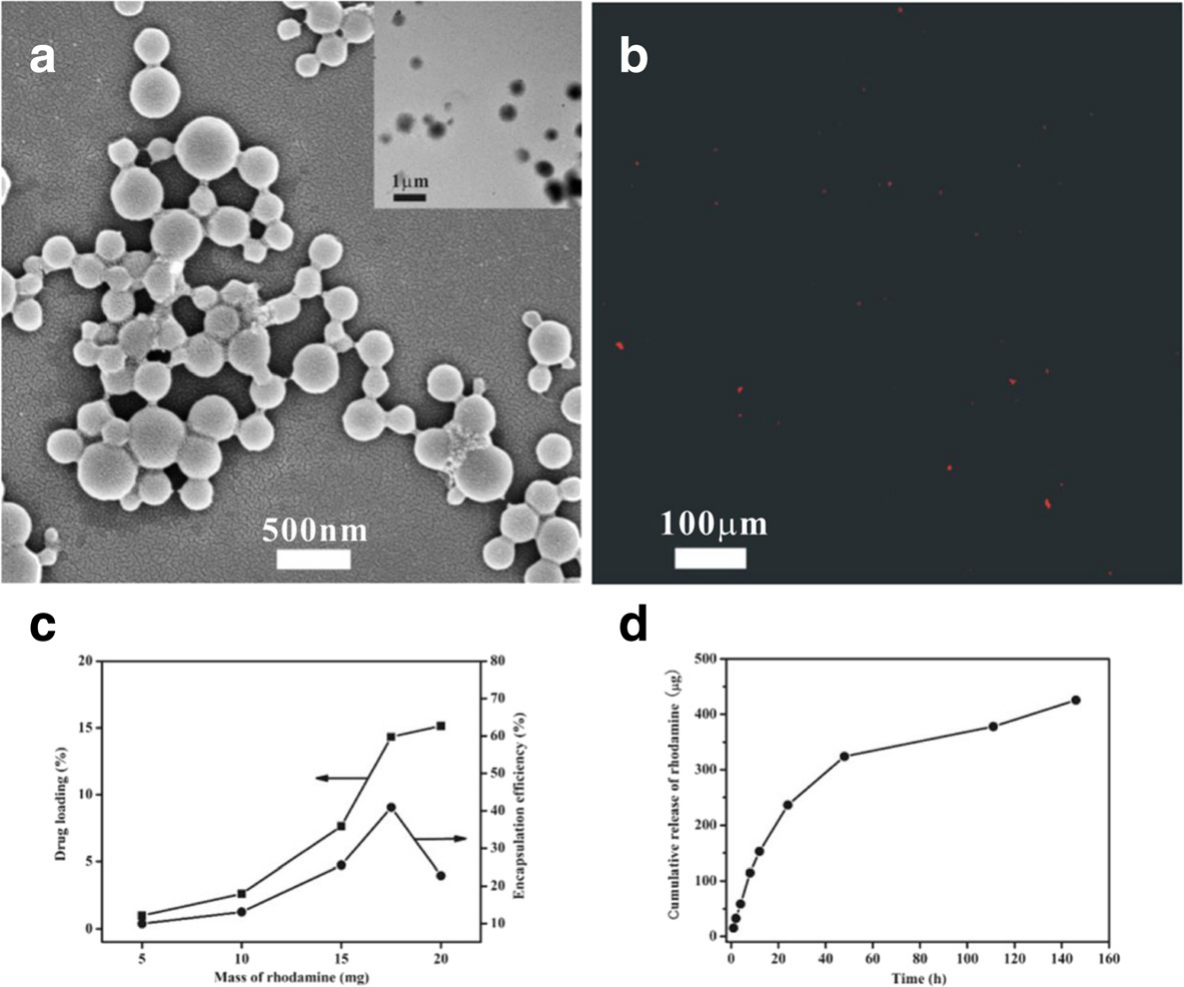
**Characteristics of RhB-loaded BSA-NPs.** SEM **(a)**, TEM (inset of (a)), and CLSM **(b)** images of RhB-loaded BSA-NPs denatured by heat are demonstrated. The drug loading capacity, encapsulation efficiency **(c)**, and controlled release profile **(d)** are shown respectively.

Rhodamine B was used as a model drug for observation and evaluation of drug loading capacity. The morphology and structure of RhB-loaded NP-H (Figure 2a) did not change in comparison with those of BSA-NPs (Figure 1a). The mean diameter of RhB-loaded NP-H was 636 nm, larger than that of BSA-NPs.

The BSA-NPs and RhB-BSA-NPs had zeta potential values of -15.4 and +4.98 mV, respectively. The potential difference demonstrated that the positively charged RhB had an interaction with the negatively charged BSA [8], which also promoted the attachment of RhB to the BSA. The fluorescent image of the RhB-BSA-NPs (Figure 2b) further confirmed that RhB had attached to the BSA-NPs. Thus, the model drug and small molecules could affect certain parameters including size and charge of polymers, which was in agreement with the previous reports [16-19].

The drug loading capacity and encapsulation efficiency of BSA-NPs were also evaluated. The drug loading capacity of BSA was 15.4% for RhB (Figure 2c). The maximum encapsulation efficiency was 40.9% (Figure 2c). It was likely attributed to the electrostatic interaction and hydrophobic interactions between RhB and BSA followed by diffusion of the model drug into the BSA matrix [8,16]. Nevertheless, the drug cannot diffuse into the matrix more after achieving the kinetic equilibrium state. The results in this report were consistent with the report described by Shi and Goh [8].

The *in vitro* drug release profile of RhB from BSA-NPs is shown in Figure 2d. A good sustained release profile is achieved. The cumulative release of RhB over a period of 150 hours was 429.14 μg, indicating a good affinity between the BSA and RhB. This was governed by Fickian diffusion due to the electrostatic interaction, which restricted the release of positively charged RhB from negatively charged BSA *in vitro*.

### *In vitro* cytocompatibility study

**Figure 3:**
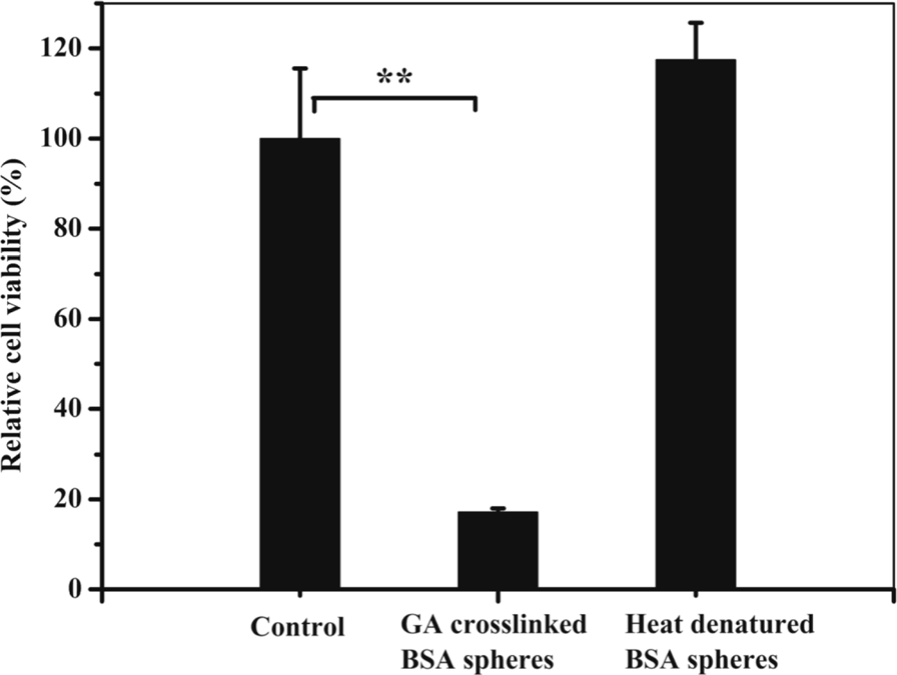
**Cytotoxity evaluation of BSA-NPs fixed by GA or denatured by heat against L929 cells.** Each value represents mean ± SD (*n* = 3) (**P < 0.01).

*In vitro* experiment of BSA-NPs cross-linked with GA or denatured by heat against L929 cell lines were performed by CCK-8 to evaluate the cytocompatibility. As shown in Figure 3, cell viability of NP-GA was significantly lower (*P* = 0.001) than that of the control possibly because the water wash in this study was only once. These results indicated that the NP-H had a better cytocompatibility than the NP-GA. The slight cytotoxicity of NP-GA was in agreement with that reported by Speer [20]. There was no statistical difference between the NP-H (*P* = 0.114) and the control.

**Figure 4:**
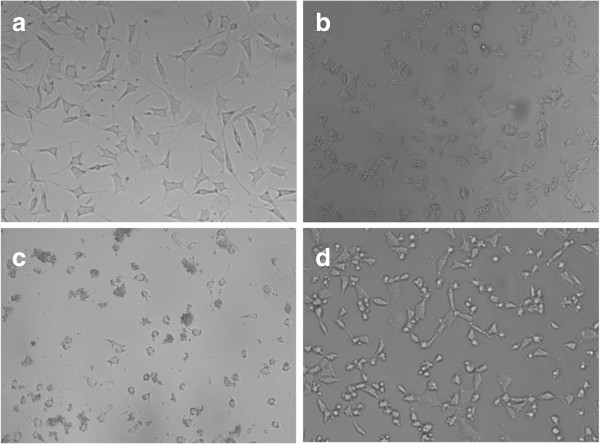
**Morphology of L929 cells cultured with different conditions.** L929 cells cultured in DMEM-10% FBS as the control **(a)**, after performing CCK-8 assay **(b)**, with the addition of NP-GA **(c)** and NP-H **(d)**, are demonstrated respectively. All images have an original magnification of × 200.

The shape of L929 cells incubated with NP-H maintained high viability after the assay (Figure 4d) while round-shaped cells could be observed in the control and NP-GA groups (Figure 4b,c). This indicated that the addition of nontoxic NP-H might provide nutrition and promote cell proliferation due to the hydrophobic domain of such natural protein, just as the silk fibroin particles did [8]. But the nutrition property of BSA on cell proliferation cannot compensate the side effect of GA in the system, which explained the fact that most cells died with the addition of NP-GA (Figure 4c). The above findings disclosed that BSA was not only a soft material with good biocompatibility but also a nutrition provider. Further studies will focus on the assessment of BSA-NP drug delivery in the treatment of inner ear disorders.

### *In vivo* distribution and drug delivery of BSA-NPs

**Figure 5:**
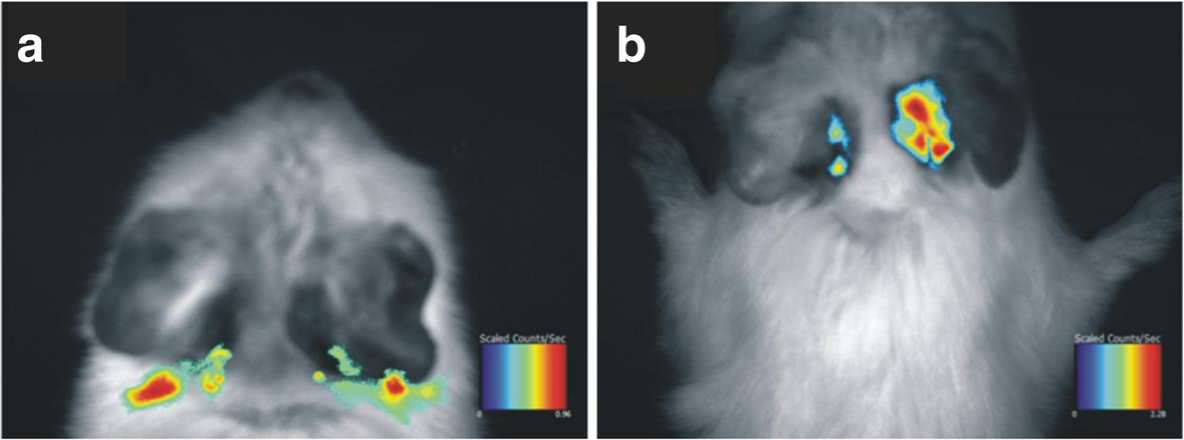
**Live images revealed the distribution of RhB-BSA-NPs.** RhB-BSA-NPs with heat denaturation were injected into the right ear, and the images were taken immediately **(a)** and 72 h later **(b)**. RhB solution injected into the left ear was the control.

As for the good cytocompatibility, BSA-NPs with heat denaturation were loaded with RhB and used to evaluate the local drug delivery. Acoustic bullae of guinea pigs with entire RWM were isolated and injected with RhB-BSA-NP (right ear) and RhB solution (left ear). The live images were taken immediately (Figure 5a). Three days later, there was still obvious fluorescent signals with a larger area in the right ear (Figure 5b), which indicated that RhB-BSA-NPs was retained nearby the RWM and RhB possibly diffused into the Eustachian tube and the inner ear. We assumed that the BSA-NPs maybe useful for local drug delivery and controlled release. In the left ear, there were only minimal fluorescent signals of RhB (Figure 5b) because the RhB could penetrate through the RWM by simple diffusion along its concentration gradient.

**Figure 6:**
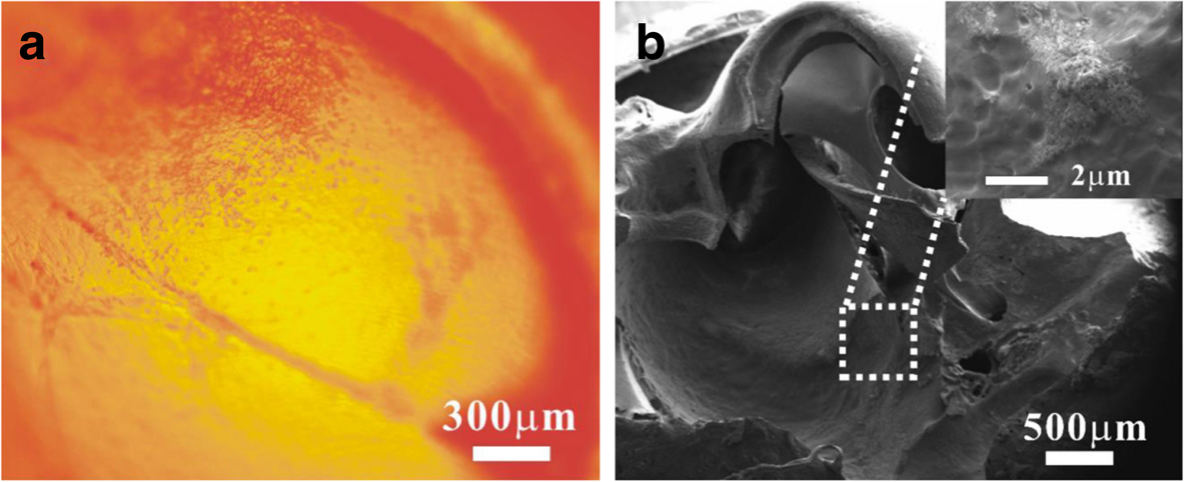
**Images of RhB-BSA-NPs adhering on the RWM and osseous spiral lamina.** The fluorescent image of RhB-BSA-NPs **(a)** adhering on the RWM was taken immediately after the surgery. The SEM image of RhB-BSA-NPs **(b)** deposited on the osseous spiral lamina was taken 3 days later. The aggregated BSA-NPs are shown in the inset (inset of (b)).

The guinea pigs were then killed and the temporal bones and RWMs were separated. The nanoparticles still attached on the RWM (Figure 6a). The SEM image revealed that particles aggregated on the osseous spiral lamina and some particles even had penetrated into the cochlea through the RWM (Figure 6b). As previously described that PLGA nanoparticles or lipid core nanocapsules could pass through the RWM and be deposited in various sites of the cochlea [5,21-23], we assumed that the tiny BSA-NPs loaded with RhB could successfully reach the inner ear through the RWM.

## Conclusions

In summary, BSA-NPs were fabricated via a desolvation method. The heat-denatured BSA-NPs had a great potential application for local drug delivery into the cochlea to treat inner ear diseases due to the tiny size, good biocompatibility, drug loading capacity, and controlled release profile. Further studies will focus on the evaluation of drug-loaded BSA-NPs, including prednisolone. We will evaluate their pharmacokinetics, pharmacodynamics, and delivery mechanism in animal model. The BSA-NPs also shed light in the treatment of human inner ear diseases.

## Abbreviations

BSA: bovine serum albumin; BSA-NP: bovine serum albumin nanoparticles; RWM: round window membrane; PLGA: polylactic/glycolic acid; RhB: rhodamine B; RhB-BSA-NP: RhB-loaded BSA-NP; GA: glutaraldehyde; CCK-8: cell counting kit-8; NP-GA: BSA-NPs with GA fixation; NP-H: BSA-NPs with heat denaturation; DLS: dynamic light scattering; TEM: transmission electron microscope; SEM: scanning electron microscopy.

## Competing interests

The authors declare that they have no competing interests.

## Authors’ contributions

ZY, ZZ, GH, QX, and MY performed the experiments and analyzed the results. ZY and MY conceived and designed the experiments, analyzed the results, and participated in writing the manuscript. All authors read and approved the final manuscript.

## Authors’ information

ZY is a professor from the Department of Otorhinolaryngology, The Second Artillery General Hospital of Chinese People's Liberation Army, Beijing, 100088, People's Republic of China, and Center of Otorhinolaryngology, Naval General Hospital of Chinese People's Liberation Army, Beijing, 100037, People's Republic of China. MY is a Ph.D. from the Department of Cell Biology, Key Laboratory of Cell Biology, Ministry of Public Health, College of Basic Medicine, China Medical University, Shenyang 110001, People's Republic of China. ZZ, GH, and QX are Ph.D. from the Institute of Biomedical Engineering, Chinese Academy of Medical Sciences & Peking Union Medical College, The Key Laboratory of Biomedical Material of Tianjin, Tianjin, 300192, People's Republic of China.
